# Advances in imaging for the diagnosis and disease monitoring of Pompe disease

**DOI:** 10.1186/1471-2474-14-S2-O2

**Published:** 2013-05-29

**Authors:** Robert-Yves Carlier

**Affiliations:** 1 Service de Radiologie, Hôpital Raymond Poincaré, Garches, France

## 

Imaging can play a role at different stages of Pompe disease. In most cases the clinical presentation and high CK level are very evocative and GAA activity testing is sufficient for the diagnosis. In some cases the clinical presentation is less typical and late onset or juvenile forms cannot be differentiated from limb girdle muscular dystrophies or congenital muscular dystrophies. In such cases previous descriptions of Whole body (WB) T1 weighted MRI images have shown very evocative patterns of most often affected or spared muscles. The most typically and frequently affected are tongue, scapulae fixators and especially sub-scapularis, trunk muscles like spine erectors, abdominal belt muscles and psoas. The most frequently spared are masticators, arm and forearms, and lower legs muscles.

In most cases the disease is associated with complications like respiratory involvement and disabling muscle weakness. Imaging is not taken into account when considering treatment options. On the contrary, when the diagnosis is made after family screening or in patients with minor symptoms, the distribution and severity of muscles involvement established by WB MRI could influence the decision to treat. Stability over time or degradation could also impact this decision. MR description of initial distribution and progression of muscle involvement could also yield a better knowledge of the disease.

Muscle biopsies are the best indicator of treatment efficacy but patient compliance could be very low if repeated follow-up biopsies were employed. Muscle imaging and especially MRI could offer the opportunity to quantify stability or progression of muscle involvement under therapy. To achieve this goal imaging has to be quantitative and not only qualitative. Quantification techniques can be developed to assess the amount of fat into muscles (DIXON) or to detect areas of muscle remodeling activity (T2 measurement). However WB MRI is useful to determine the areas where the quantification should be performed. In totally destroyed or spared muscles quantified imaging is not very pertinent. Other imaging modalities could also be used to detect regional fat increase. DEXA scanner is not only able to detect regional bone mineral density variations but also lean mass (muscles) and fat.

Muscle imaging is not always the clue for the diagnosis but it can help for recognition of atypical clinical presentations, for understanding the natural history of the disease, for the determination of patients suited for treatment as a complement of other clinical and nonclinical parameters. Medical imaging techniques of quantification could help to analyze treatment efficacy.

